# Comparative analysis of the complete chloroplast genome sequences in psammophytic *Haloxylon* species (Amaranthaceae)

**DOI:** 10.7717/peerj.2699

**Published:** 2016-11-10

**Authors:** Wenpan Dong, Chao Xu, Delu Li, Xiaobai Jin, Ruili Li, Qi Lu, Zhili Suo

**Affiliations:** 1 State Key Laboratory of Systematic and Evolutionary Botany, Institute of Botany, Chinese Academy of Sciences, Beijing, China; 2 Peking-Tsinghua Center for Life Sciences, Academy for Advanced Interdisciplinary Studies, Peking University, Beijing, China; 3 University of Chinese Academy of Sciences, Beijing, China; 4 Gansu Desert Control Research Institute, Gansu, China; 5 Beijing Botanical Garden, Institute of Botany, Chinese Academy of Sciences, Beijing, China; 6 Institute of Desertification Studies, Chinese Academy of Forestry, Beijing, China

**Keywords:** Chloroplast genome, Psammophytes, Structure, Evolution, Amaranthaceae, *Haloxylon*

## Abstract

The *Haloxylon* genus belongs to the Amaranthaceae (formerly Chenopodiaceae) family. The small trees or shrubs in this genus are referred to as the King of psammophytic plants, and perform important functions in environmental protection, including wind control and sand fixation in deserts. To better understand these beneficial plants, we sequenced the chloroplast (cp) genomes of *Haloxylon ammodendron* (HA) and *Haloxylon persicum* (HP) and conducted comparative genomic analyses on these and two other representative Amaranthaceae species. Similar to other higher plants, we found that the *Haloxylon* cp genome is a quadripartite, double-stranded, circular DNA molecule of 151,570 bp in HA and 151,586 bp in HP. It contains a pair of inverted repeats (24,171 bp in HA and 24,177 bp in HP) that separate the genome into a large single copy region of 84,214 bp in HA and 84,217 bp in HP, and a small single copy region of 19,014 bp in HA and 19,015 bp in HP. Each *Haloxylon* cp genome contains 112 genes, including 78 coding, 30 tRNA, and four ribosomal RNA genes. We detected 59 different simple sequence repeat loci, including 44 mono-nucleotide, three di-nucleotide, one tri-nucleotide, and 11 tetra-nucleotide repeats. Comparative analysis revealed only 67 mutations between the two species, including 44 substitutions, 23 insertions/deletions, and two micro-inversions. The two inversions, with lengths of 14 and 3 bp, occur in the *pet*A-*psb*J intergenic region and *rpl*16 intron, respectively, and are predicted to form hairpin structures with repeat sequences of 27 and 19 bp, respectively, at the two ends. The ratio of transitions to transversions was 0.76. These results are valuable for future studies on *Haloxylon* genetic diversity and will enhance our understanding of the phylogenetic evolution of Amaranthaceae.

## Introduction

The eudicot clade comprises approximately 75% of all flowering land plant species, including major subclades: rosids, asterids, Saxifragales, Santalales, and Caryophyllales (APG III, 2009). *Haloxylon* species, which include psammophytic small trees or shrubs, are positioned phylogenetically in the Amaranthaceae Juss of the Caryophyllales Perleb among core eudicots (APG III, 2009; Pyankov et al., 2001; Akhani, Edwards & Roalson, 2007). The *Haloxylon* genus has about 11 species, with a distribution from the Mediterranean through Central Asia and into China (Zhu, Mosyakin & Clemants, 2004). Two *Haloxylon* species, which are known as the King of psammophytic plants, are found in the deserts of northwest China and, play important roles in environmental protection, including wind control and sand fixation (Zhu, Mosyakin & Clemants, 2004; Jia & Lu, 2004). These precious psammophytic woody plants can adapt to harsh environmental conditions, such as drought, desert, high temperature, and sand storms. However, populations of *Haloxylon* plants have been threatened in China in past decades as a result of decreased underground water, overgrazing, and over exploitation of agriculture.

Because of the environmental significance of these plants and their declining numbers, genetic research on *Haloxylon* germplasm resources has garnered significant interest (Song & Jia, 2000; Sheng et al., 2004; Sheng et al., 2005; Zhang et al., 2006a; Zhang et al., 2006b). However, *Haloxylon* plants possess only fine green assimilating shoots, without leaves, making the evaluation of their phenotypic diversity difficult. Further, the detection of genetic diversity within *Haloxylon* germplasm resources has been slowed by a lack of morphological markers (Sheng et al., 2004; Sheng et al., 2005; Zhang et al., 2006a; Zhang et al., 2006b; Wang et al., 2009; Suo et al., 2012a). A recent study by Long et al. (2014) used RNA-seq data to elucidate the *Haloxylon* transcriptome, providing a valuable sequence resource for further genetic and genomic studies; however, genetic information for members of the *Haloxylon* genus, and how they might differ from one another, is limited.

Each leaf cell of plants contains 1,000–10,000 chloroplasts (cp), which are key organelles for photosynthesis and other biochemical pathways such as the biosynthesis of starch, fatty acids, pigments, and amino acids (Dong et al., 2013b; Raman & Park, 2016). Since the first cp genome of *Nicotiana tabacum* was sequenced in 1986, around 800 complete cp genome sequences have been made available in the National Center for Biotechnology Information organelle genome database. These data are valuable sources of genetic markers for phylogenetic analyses, genetic diversity evaluation, and plant molecular identification (Dong et al., 2012; Dong et al., 2013a; Dong et al., 2013b; Dong et al., 2014; Ni et al., 2016; Suo et al., 2012b).

There are two published complete cp genome sequences (*Spinacia oleracea* and *Beta vulgaris* subsp. *vulgaris*) from members of the Amaranthaceae family (Li et al., 2014; Schmitz-Linneweber et al., 2001). However, the determination of the cp genome from *Haloxylon* plants is of further significance for potentially enhancing our understanding of their adaptability to severe desert environmental conditions, and their genomic evolution within the Amaranthaceae. Here, we report the complete cp genomes from two *Haloxylon* species, *H. ammodendron* and *H. persicum*, including patterns of nucleotide substitutions, microstructural mutation, and simple sequence repeats (SSRs). We further performed genomic comparative analyses on these and two other representative Amaranthaceae species, to better understand the evolutionary relationships within this family.

## Materials & Methods

### Sampling and DNA extraction

Fresh young shoots of *H. ammodendron* (HA) and *H. persicum* (HP) were collected in May 2011 from Minqin Eremophytes Botanical Garden (N38°34′, E102°59′, Altitude 1,378 m), Gansu Province, China (under the leadership of Gansu Desert Control Research Institute, Lanzhou, Gansu, China). These HA and HP plants were originally introduced from the Turpan Desert Botanical Garden of Chinese Academy of Sciences, Xinjiang Uygur Autonomous Region. The shoots from each accession were immediately dried using silica gel for future DNA extraction. Total genomic DNA was extracted from each using the Plant Genomic DNA Kit (DP305) from Tiangen Biotech (Beijing) Co., Ltd., China. The approval numbers are 2012BAD16B0101 and 80117B1001 for field permit of the research.

### Chloroplast genome sequencing

The HA and HP cp genomes were sequenced using the short-range PCR method reported by Dong et al. (2012), Dong et al. (2013a) and Dong et al. (2013b). The PCR protocol was as follows: preheating at 94 °C for 4 min, 34 cycles at 94 °C for 45 s, annealing at 55 °C for 40 s, and elongation at 72 °C for 1.5 min, followed by a final extension at 72 °C for 10 min. PCR amplification was performed in an Applied Biosystems VeritiTM 96-Well Thermal Cycler (Model#: 9902, made in Singapore). The amplicons were sent to Shanghai Majorbio Bio-Pharm Technology Co., Ltd. (Beijing) for Sanger sequencing in both the forward and reverse directions using a 3730xl DNA analyzer (Applied Biosystems, Foster City, CA, USA). DNA regions containing poly structures or that were difficult to amplify were further sequenced using newly designed primer pairs for confirming reliable and high quality sequencing results.

### Chloroplast genome assembling and annotation

The cp DNA sequences were manually confirmed and assembled using Sequencher (v4.6) software, and cp genome annotation was performed using the Dual Organellar Genome Annotator (DOGMA) (Wyman, Jansen & Boore, 2004). BLASTX and BLASTN searches were utilized to accurately annotate the protein-encoding genes and to identify the locations of the transfer RNAs (tRNAs) and ribosomal RNAs (rRNAs). Gene annotation information from other closely related plant species was also used for confirmation when the boundaries of the introns or exons could not be precisely determined because of the limited power of BLAST in cp genome annotation (e.g., for some short exons of 6–9 nt in length, such as in the case of *rps*16, *pet*B, and *pet*D). Promoter, intron, and exon boundaries, as well as the location of stop codons for all protein-encoding genes, have been identified accurately. The cp genome map was drawn using Genome Vx software (Conant & Wolfe, 2008) (http://wolfe.ucd.ie/GenomeVx/), and the cp genome sequences have been deposited to GenBank with the following accession numbers: KF534478 for HA and KF534479 for HP (https://www.ncbi.nlm.nih.gov/nuccore/?term=Haloxylon+chloroplast+genome).

### Repeat structure analysis

Gramene **Simple Sequence Repeat Identification Tool** software (http://www.gramene.org/db/markers/ssrtool) (Benson, 1999) was utilized to search for simple sequence repeat loci in the cp genome sequences, with the threshold value of repeat number as ≥10 for mono-nucleotide repeats, ≥5 for di-nucleotide repeats, ≥4 for tri-nucleotide repeats, and ≥3 for tetra-nucleotide, penta-nucleotide, or hexa-nucleotide repeats.

### Gene content analysis and comparative genomics

The mVISTA program was employed in Shuffle-LAGAN mode (Frazer et al., 2004) to compare the complete HA and HP cp genomes. These were aligned using MUSCLE software (Thompson et al., 1997) and were manually adjusted using Se-Al 2.0 (Rambaut, 1996). Variable sites in the cp genome were calculated using DnaSP (DNA Sequences Polymorphism version 5.10.01) software (Librado & Rozas, 2009), and the genetic distance (*p*-distance) was computed using MEGA 6.0 software (Tamura et al., 2013). Based on the aligned sequence matrix, the micro-structure events were checked manually and were further divided into three categories: (i) microsatellite-related insertions/deletions (indels), (ii) non-microsatellite-related indels, (iii) and inverted sequences. Using the HA cp genome sequence as the standard reference, the size, location, and evolutionary direction of the microstructure events were counted. The proposed secondary structures of the inverted regions in the cp genomes of HA and HP were analyzed using mfold software (Zuker, 2003). The complete cp genome sequences of *S. oleracea* (GenBank accession number AJ400848.1, *Spinacia* L.) (Schmitz-Linneweber et al., 2001) and *B. vulgaris* subsp. *vulgaris* (GenBank accession number KJ081864.1, *Beta vulgaris* subsp. *vulgaris*) (Li et al., 2014), two closely related species in the Amaranthaceae family, were downloaded from GenBank databases (www.ncbi.nlm.nih.gov). These were used for comparison with the complete cp genomes of HA and HP.

## Results & Discussion

### Genome features

**Figure 1:**
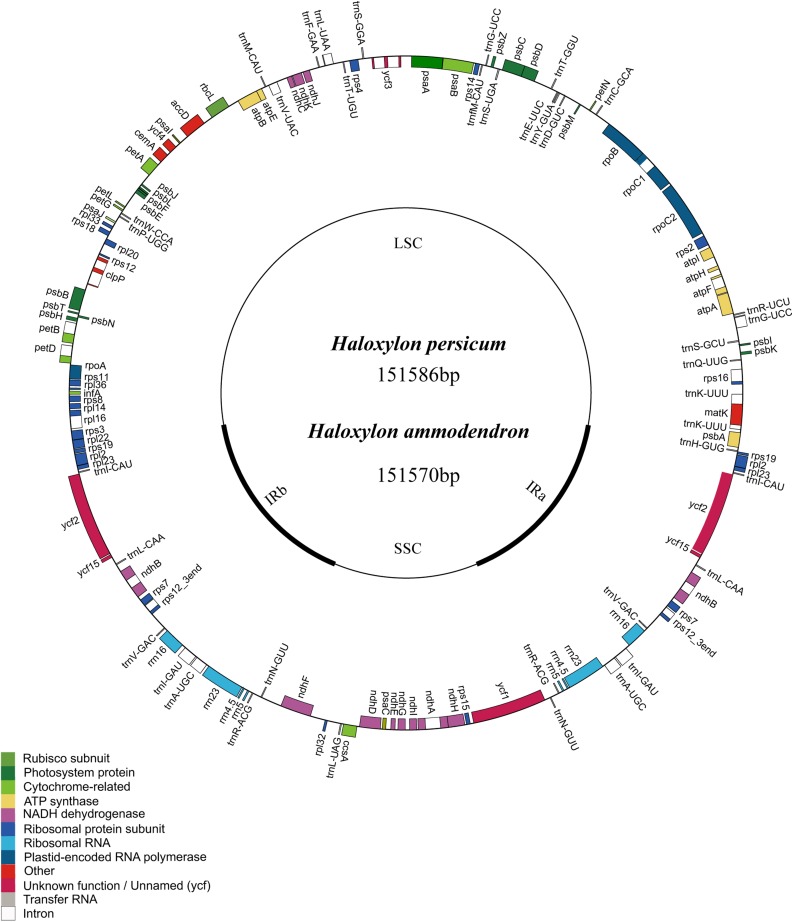
Representative map of the two *Haloxylon* chloroplast genomes. Genome annotation was performed using DOGMA. Genes drawn outside of the circle are transcribed clockwise, whereas those represented inside the circle are transcribed counterclockwise. Small single copy (SSC), large single copy (LSC), and inverted repeat (IRa, IRb) regions are indicated.

Similar to the typical cp genome structure in other higher plants, the *Haloxylon* cp genome is a double-stranded, circular DNA molecule of 151,570 bp in length in HA and 151,586 bp in length in HP. It also includes a large single copy region (LSC) of 84,214 bp in HA and 84,217 bp in HP and a small single copy region (SSC) of 19,014 bp in HA and 19,015 bp in HP; these are separated by a pair of inverted repeats (IR) (24,171 bp in HA and 24,177 bp in HP) (Fig. 1). The GC content in this IR region is 43.0% in HA and 42.7% in HP, and the GC content in the LSC and SSC regions is 34.4% (LSC) and 29.7% (SSC) in HA and 34.5% (LSC) and 29.7% (SSC) in HP (Table 1).

**Table 1:** Summary of complete chloroplast genome features in *Haloxylon*.

	*H. ammodendron*	*H. persicum*	*Spinacia oleracea*	*Beta vulgaris*
Total cpDNA size	151,570	151,586	150,725	149,635
Length of LSC region	84,214	84,217	82,719	83,057
Length of IR region	24,171	24,177	25,073	24,439
Length of SSC region	19,014	19,015	17,860	17,701
Total GC content (%)	36.6	36.6	36.9	36.4
LSC	34.4	34.5	34.8	34.1
IR	43.0	43.0	42.7	42.2
SSC	29.7	29.7	29.8	29.2
Total number of genes	112	112	112	113
Protein encoding	78	78	78	79
tRNA	30	30	30	30
rRNA	4	4	4	4
Pseudogenes	2	2	2	1

Among the four Amaranthaceae species included in our analyses, which represent three genera, the longest cp genomes (151,570 bp for HA and 151,586 bp for HP) are 1,935 bp to 1,951 bp larger than the shortest one (149,635 bp for *B. vulgaris* subsp. *vulgaris*) (Li et al., 2014). The size of the *S. oleracea* cp genome (150,725 bp) (Schmitz-Linneweber et al., 2001) is intermediate (Table 1). Notably, the cp genomes of HP and HA are quite similar in size; the HP cp is only 16 bp longer than that of HA, with minor differences between them.

There are a total of 112 genes in the *Haloxylon* cp genome, including 78 coding genes, 18 of which are duplicated genes in the IR region, 30 tRNA genes, and four ribosomal RNA genes (16S, 23S, 5S, 4.5S) (Fig. 1 and Table S1). Based on their predicted functions, these genes can be divided into three categories, (1) genes related to transcription and translation; (2) genes related to photosynthesis; (3) genes related to the biosynthesis of amino acids, fatty acids, etc., and some functionally unknown genes (Table S1). The *S. oleracea* cp also contains the same 78 protein-coding genes, whereas the cp in *B. vulgaris* has 79. This species contains an additional gene (*rpl*23), which is a pseudogene in the other species (Fig. 1 and Table S1). There are 17 genes harboring introns in the cp genomes of the four Amaranthaceae species analyzed (one class I intron, *trn*L-UUA, and 16 class II introns), and two of these genes, *ycf*3 and *clp*P, contain two introns each (Table 2).

**Table 2:** Genes with introns in *Haloxylon ammodendron* and *H. persicum* and length of exons and introns.

	Exon I (bp)	Intron I	Exon II	Intron II	Exon III
*atp*F	145(145)	785(784)	410(410)		
*clp*P	71(71)	951(951)	292(292)	601(601)	228(228)
*ndh*A	553(553)	1090(1090)	533(533)		
*ndh*B	777(777)	675(675)	756(756)		
*pet*B	6(6)	801(801)	642(642)		
*pet*D	8(8)	722(722)	475(475)		
*rpl*16	399(399)	913(913)	9(9)		
*rpl*2	393(393)	668(668)	435(435)		
*rpo*C1	432(432)	780(780)	1602(1602)		
*rps*12	114(114)	–	231(231)	–	27(27)
*rps*16	40(40)	881(881)	197(197)		
*trn*A-UGC	38(38)	831(831)	42(42)		
*trn*G-GCC	23(23)	722(722)	58(58)		
*trn*I-GAU	42(42)	942(941)	35(35)		
*trn*K-UUU	35(35)	2909(2909)	37(37)		
*trn*L-UAA	35(35)	557(557)	50(50)		
*trn*V-UAC	39(39)	602(602)	35(35)		
*ycf*3	126(126)	772(772)	229(229)	812(812)	152(152)

**Notes.**

*rps*12 is trans-spliced with the 5′end located in the LSC region and the duplicated 3′end in the IR regions.

Several angiosperm lineages have lost introns from the *rpl*2 gene independently (Downie et al., 1991), which could also be regarded as a characteristic feature of the core members of the Caryophyllales (Logacheva et al., 2008). In each of the four Amaranthaceae cp genomes in our analysis, the *rpl*2 gene has lost its intron. Some authors have proposed that intron loss is not always a dependable marker of phylogenetic relationships (Millen et al., 2001; Dong et al., 2013b; Raman & Park, 2016), and further study, including the sampling of more taxa, is needed to clarify this issue.

### Expansion and contraction of the border regions in *Haloxylon* cp genomes

**Figure 2:**
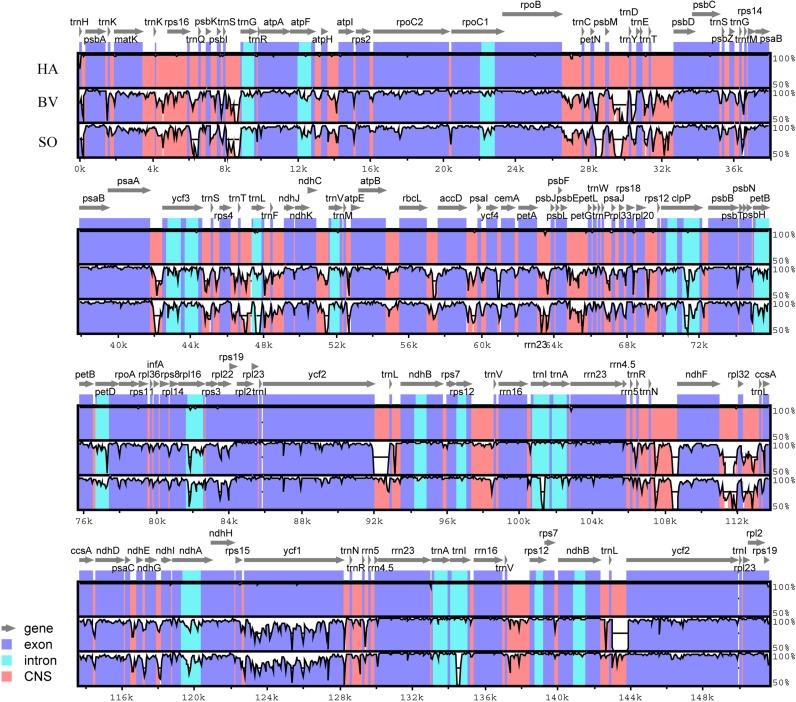
Identity plot comparing the chloroplast genomes of four Amaranthaceae species using *Haloxylon ammodendron* as a reference sequence. The vertical scale indicates the percent identity, ranging from 50% to 100%. The horizontal axis indicates the coordinates within the chloroplast genome. Genomic regions are color coded as protein-coding, rRNA, tRNA, intron, and conserved non-coding sequences (CNS). Abbreviations HP, *H. persicum*; SO, *Spinacia oleracea*; BV, *Beta vulgaris* subsp. *vulgaris*.

To analyze these Amaranthaceae species at the genome-level, the sequences of all the four cp genomes were plotted using the VISTA program (Frazer et al., 2004), using the annotation of HA as a reference (Fig. 2). Similar to other angiosperms, we observed that the IR region is more conserved in these species than the LSC and SSC regions.

**Figure 3:**
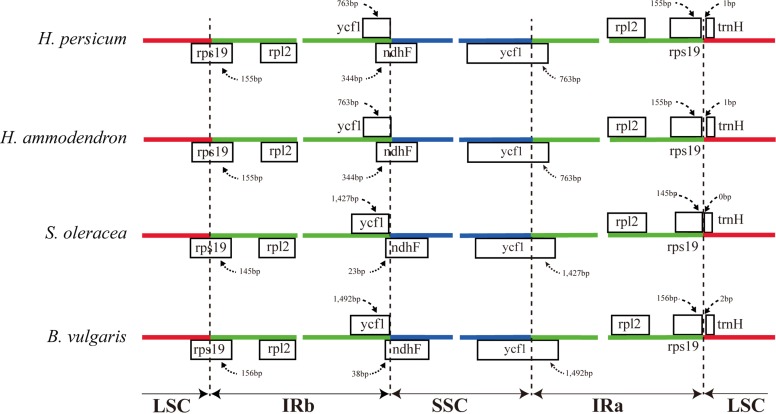
Comparison of the junction positions between the single copy and IR regions among four Amaranthaceae genomes.

The expansion and contraction of the border regions between the two IR regions and the single copy region have contributed to genome size variations among plant lineages (Dong et al., 2013b; Goremykin et al., 2003; Ni et al., 2016). Therefore, we next compared the exact IR border positions and their adjacent genes among the four Amaranthaceae cp genomes (Fig. 3). From these data, we see that the IRa/LSC border is generally located upstream of the *trn*H-GUG gene. The distance between the IRa/LSC border and the *trn*H-GUG gene is 1 bp in the *Haloxylon* cp genomes and 2 bp in *Beta* genus, with no separation in *Spinacia* (Fig. 3). The IR region is expanded by 763 bp and enters the 5′ end of the *ycf*1 gene in *Haloxylon* species, whereas it is expanded by 1,427 bp and 1,492 bp, respectively, in *Spinacia* and *Beta*. Except for the expansion of the *ycf*1 gene, the IR region extends to the *rps*19 gene in all of four Amaranthaceae cp genomes. The *rps*19 pseudogene was not observed in this study. Although there are expansions or contractions of IR regions observed among the investigated species of the Amaranthaceae, they contribute little to the overall size differences in the cp genomes. The exon at the 5′ end of the *rps*12 gene is located in the LSC region, and the intron and 3′-end exon of the gene are situated in the IR region in all four Amaranthaceae species.

### Indels and SNPs

Indel and single nucleotide polymorphism (SNP) sites are important molecular features valuable for development of DNA markers that are useful for plant identification and genetic analysis of population structure (Dong et al., 2012; Dong et al., 2013a; Dong et al., 2013b; Dong et al., 2014; Suo et al., 2012b; Suo et al., 2015; Suo et al., 2016). We detected 23 indels in the cp genome sequence alignment of HA and HP, including 16 indels caused by microsatellite repeat variations and seven non-microsatellite-related indels (Table 3). Most of the indel events occurred in non-coding regions (21/23). A large portion of the indels related to microsatellite repeat variations are characterized by a single base mutation; six insertions of this type were observed in the HA cp genome. The non-microsatellite-related indels were found to contain mostly five to six variable base sites, and two insertions of this type were detected in the HA cp genome.

**Table 3:** Indel mutation events in the chloroplast genomes of *Haloxylon ammodendron* and *H. persicum*.

Region	Location	Types	HA	HP	Length (bp)	Direction^a^
*acc*D-*psa*I	Intergenic	Homopolymeric indel	A A	–	2	Insertion
*atp*A-*atp*F	Intergenic	Homopolymeric indel	T	–	1	Insertion
*atp*F	Intron	Homopolymeric indel	–	T	1	Deletion
*ndh*I-*ndh*A	Intergenic	Homopolymeric indel	–	A	1	Deletion
*ndh*J-*ndh*K	Intergenic	Homopolymeric indel	–	T	1	Deletion
*psb*I-*trn*S	Intergenic	Homopolymeric indel	–	T	1	Deletion
*psb*I-*trn*S	Intergenic	Homopolymeric indel	–	A	1	Deletion
*rbc*L-*acc*D	Intergenic	Homopolymeric indel	–	A	1	Deletion
*rps*18-*rpl*20	Intergenic	Homopolymeric indel	T	–	1	Insertion
*trn*E-*trn*T	Intergenic	Homopolymeric indel	–	A	1	Deletion
*trn*K-*rps*16	Intergenic	Homopolymeric indel	A	–	1	Insertion
*trn*K-*rps*16	Intergenic	Homopolymeric indel	A	–	1	Insertion
*trn*L	Intron	Homopolymeric indel	–	A	1	Deletion
*trn*L	Intron	Homopolymeric indel	A	–	1	Insertion
*trn*L	Intron	Homopolymeric indel	–	T	1	Deletion
*trn*R-*apt*A	Intergenic	Homopolymeric indel	–	T	1	Deletion
*atp*H-*atp*I	Intergenic	Indel	T T T A T T	–	5	Insertion
*clp*P-*psb*B	Intergenic	Indel	–	**G** T C T T	5	Deletion
*pet*L-*pet*G	Intergenic	Indel	–	**G**	1	Deletion
*rpo*B-*trn*C	Intergenic	Indel	–	T **G** T A T T	5	Deletion
*rpo*B-*trn*C	Intergenic	Indel	T A C A A	–	5	Insertion
*rrn*23	Coding	Indel	–	A A T T A A	6	Deletion
*rrn*23	Coding	Indel	–	T T A A T T	6	Deletion

**Notes.**

a The chloroplast genome of *H. ammodendron* was used as a standard.

HA *H. ammodendron* HP *H. persicum*

**Table 4:** The nucleotide substitution patterns present in the two *Haloxylon* chloroplast genomes.

Region	Location	*H. ammodendron*	*H. persicum*
*atp*A	Coding	**G**	A
*atp*I	Coding	T	C
*mat*K	Coding	C	A
*ndh*F	Coding	C	T
*ndh*I	Coding	**G**	T
*psb*C	Coding	A	C
*rpo*B	Coding	C	T
*rpo*C2	Coding	C	A
*rpo*C2	Coding	C	**G**
*rpo*C2	Coding	**G**	T
*rps*15	Coding	A	**G**
*rps*3	Coding	T	**G**
*ycf*1	Coding	A	**G**
*ycf*1	Coding	**G**	C
*ycf*1	Coding	**G**	T
*atp*B-*rbc*L	Intergenic	A	C
*atp*F-*atp*H	Intergenic	**G**	C
*atp*H-*atp*I	Intergenic	**G**	A
*ndh*F-*rpl*32	Intergenic	**G**	T
*psa*J-*rpl*33	Intergenic	C	T
*psa*J-*rpl*33	Intergenic	T	A
*psb*E-*pet*L	Intergenic	C	A
*psb*M-*trn*D	Intergenic	A	**G**
*rpl*14-*rpl*16	Intergenic	T	**G**
*rpl*20-*rps*12	Intergenic	**G**	T
*rpl*33-*rps*18	Intergenic	T	C
*rpo*A-*rps*11	Intergenic	A	**G**
*rpo*A-*rps*11	Intergenic	T	C
*rpo*B-*trn*C	Intergenic	**G**	T
*rpo*B-*trn*C	Intergenic	T	**G**
*rps*18-*rpl*20	Intergenic	T	**G**
*rps*8-*rpl*14	Intergenic	**G**	A
*trn*G-*trn*R	Intergenic	A	C
*trn*H-*psb*A	Intergenic	T	**G**
*trn*K-*mat*K	Intergenic	A	C
*trn*K-*rps*16	Intergenic	A	C
*trn*P-*psa*J	Intergenic	C	T
*trn*P-*ps*aJ	Intergenic	C	T
*clp*P	Intron	T	**G**
*ndh*A	Intron	T	C
*rpl*16	Intron	T	C
*rps*16	Intron	T	**G**
*trn*V	Intron	T	C
*ycf*3	Intron	T	C

Forty-four SNPs were detected in the HA and HP cp genomes (Table 4), which is considerably less than what was found between the cp genomes of other closely related plant species, including *Oryza sativa* and *Oryza nivara* (159 SNPs, Masood et al., 2004), *Machilus yunnanensis* and *Machilus balansae* (231 SNPs, Song et al., 2015), *Citrus sinensis* and *Citrus aurantiifolia* (330 SNPs, Su et al., 2014), *Panax ginseng* and *Palax notoginseng* (464 SNPs, Dong et al., 2014), and *Solanum tuberosum* and *Solanum bulbocastanum* (591 SNPs, Chung et al., 2006). Of note, the indel and SNP mutation events in the *Haloxylon* cp genomes were not randomly distributed, but rather, clustered as “hotspots” (Shaw et al., 2007; Worberg et al., 2007). It is likely that such mutational dynamics created the highly variable regions in the genome (Suo et al., 2012b; Song et al., 2015).

### Patterns of nucleotide substitutions

Overall, the differences between the HA and HP cp genomes are minor, with a genetic distance of 0.00029 between them (Table 4). In total, 44 variable nucleotide sites were detected, 23 of which were found in intergenic regions, six in introns, and 15 in protein-encoding regions.

**Figure 4:**
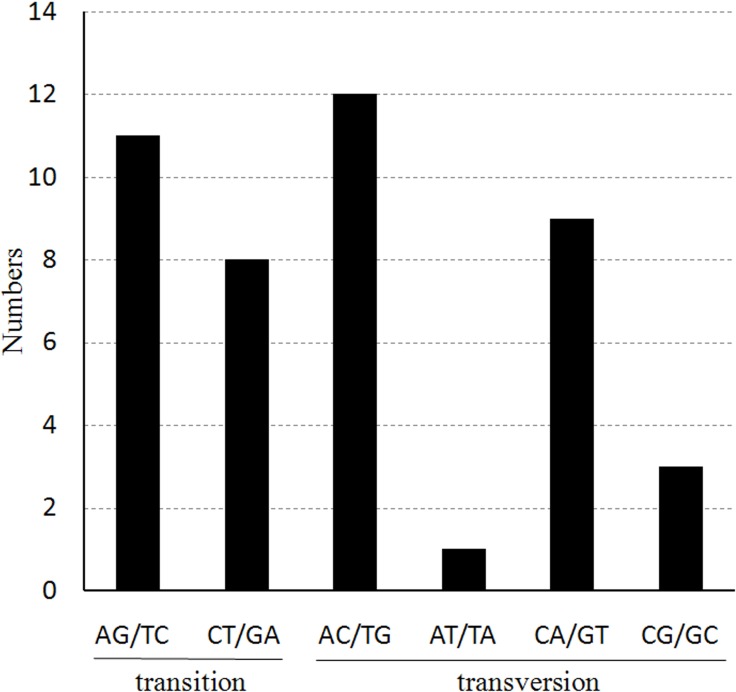
The nucleotide substitution patterns in the two *Haloxylon* chloroplast genomes. The patterns were divided into six types, as indicated by the six non-strand-specific base-substitution types (i.e., numbers of G to A and C to T sites for each respective set of associated mutation types). The *H. ammodendron* chloroplast genome was used as a standard.

We also found that the probability of occurrence for the various nucleotide substitutions is different, depending on the mutation, as shown in Fig. 4. The most frequently occurring mutations are from A to C and from T to **G** (12 times each); mutations from A to T and from T to A exhibited the lowest frequency (only one occurrence of each). The ratio of transitions (Ts) and transversions (Tv) was 0.76 in the cp genome of *Haloxylon* species.

In the gene-encoding regions of the HA and HP cp genomes, a total of 15 variable base sites were detected in 11 protein-encoding genes. Specifically, we found one mutation in each of the following genes: *atp*A, *atp*I, *mat*K, *ndh*F, *ndh*I, *psb*C, *rpo*B, *rps*15, and *rps*3. Two genes, * rpo*C2 and *ycf*1, each contained three mutation sites (Table 5). These mutations included six Ts and nine Tv. Ten nonsynonymous substitutions occurred simultaneously in seven genes (Table 5).

**Table 5:** Comparison of the mutational changes, number of transitions (*Ts*) and transversions (*Tv*), and synonymous (*S*) and nonsynonymous (*N*) substitutions per protein-coding chloroplast gene in *Haloxylon ammodendron* and *H. persicum*.

Gene	*Ts*	*Tv*	*S*	*N*
* atp*A	1	0	1	0
*atp*I	1	0	1	0
*mat*K	0	1	0	1
* ndh*F	1	0	0	1
*ndh*I	0	1	0	1
* psb*C	0	1	1	0
*rpo*B	1	0	1	0
*rpo*C2	0	3	0	3
*rps*15	1	0	0	1
*rps*3	0	1	0	1
*ycf*1	1	2	1	2
Total	6	9	5	10

**Table 6:** Location of repeats in the *Haloxylon ammodendron* chloroplast genome.

No.	Location	Motif	No. of repeats	SSR start	SSR end
1	*trn*K-*mat*K	A	11	1,658	1,668
2	*trn*K-*rps*16	A	12	4,210	4,221
3	*rps*16-*trn*Q	A	10	6,461	6,470
4	*trn*Q-*psb*K	A	10	6,957	6,966
5	*psb*K-*psb*I	A	10	7,578	7,587
6	*psb*I-*trn*S	A	12	7,854	7,865
7	*atp*F intron	A	10	12,476	12,485
8	*rpo*C1 intron	A	10	22,386	22,395
9	*trn*E-*trn*T	A	10	31,169	31,178
10	*trn*L-intron	A	12	47,464	47,475
11	*trn*F-*ndh*J	A	10	48,982	48,991
12	*rbc*L-*acc*D	A	12	57,323	57,334
13	*acc*D-*psa*I	A	10	59,584	59,593
14	*psb*F	A	10	64,309	64,318
15	*clp*P intron	A	10	71,717	71,726
16	*pet*B intron	A	18	75,505	75,522
17	*ndh*I-*ndh*A	A	10	118,705	118,714
18	*psa*A	C	10	40,165	40,174
19	*trn*K-*rps*16	T	10	4,464	4,473
20	*psb*I-*trn*S	T	10	7,745	7,754
21	*trn*R-*atp*A	T	11	9,948	9,958
22	*atp*A-*atp*F	T	10	11,532	11,541
23	*atp*F intron	T	11	12,457	12,467
24	*rps*2-*rpo*C2	T	11	15,957	15,967
25	*rps*2-*rpo*C2	T	11	18,156	18,166
26	*rpo*B	T	10	25,865	25,874
27	*trn*D-*trn*Y	T	10	30,323	30,332
28	*trn*L-*trn*F	T	10	48,029	48,038
29	*ndh*J-*ndh*K	T	10	49,646	49,655
30	*trn*V intron	T	15	52,214	52,228
31	*trn*M-*atp*E	T	10	52,658	52,667
32	*rbc*L-*acc*D	T	14	57,377	57,390
33	*pet*L-*pet*G	T	10	66,141	66,150
34	*psa*J-*rpl*33	T	12	67,499	67,510
35	*rps*18-*rpl*20	T	10	68,447	68,456
36	*rpo*A	T	10	78,219	78,228
37	*rps*11-*rpl*36	T	12	79,577	79,588
38	*rpl*32-*trn*L	T	11	11,2371	11,2381
39	*ndh*A intron	T	12	119,581	119,592
40	*ndh*A intron	T	10	119,793	119,802
41	*ycf*1	T	12	125,285	125,296
42	*ycf*1	T	10	125,890	125,899
43	*ycf*1	T	14	126,895	126,908
44	*ycf*1	T	10	127,195	127,204
45	*rps*16-*trn*Q	A T	5	6,277	6,286
46	*trn*S-*trn*G	A T	5	8,177	8,186
47	*trn*S-*trn*G	A T	5	8,300	8,309
48	*trn*N-*ndh*F	T A A	4	109,380	109,391
49	*psb*A-*trn*K	T T **G** T	3	1,522	1,533
50	*mat*K-*trn*K	T T C T	3	3,873	3,884
51	*atp*I-*rps*2	A T T A	3	15,121	15,132
52	*trn*E-*trn*Y	A T T A	3	31,084	31,095
53	*acc*D-*psa*I	T A A T	4	59,721	59,736
54	*rps*18-*rpl*20	T T T A	3	68,474	68,485
55	*clp*P intron	T T T C	3	71,598	71,609
56	*rrn*23	A **G** **G** T	3	104,481	104,492
57	*trn*L-*ccs*A	A A C C	3	113,312	113,323
58	*ycf*1	T A A T	3	124,297	124,308
59	*rrn*23	C TA C	3	131,310	131,321

### Repeat structure feature

Simple sequence repeats (SSRs) are also called microsatellites. Within the cp genomes of HA and HP, 59 different SSR loci were detected. Of these, 44 loci are mono-nucleotide repeats, three are di-nucleotide repeats, one is a tri-nucleotide repeat, and 11 are tetra-nucleotide repeats; penta-nucleotide repeats or those containing a higher number of nucleotide repeats were not detected. Among the SSR loci detected, the most frequently observed repeats were A/ T and A T/ T A, accounting for 77.97% of the total number of SSR loci (Table 6). By comparison, in the cp genomes of *M. yunnanensis* and *M. balansae*, 36 SSR loci were identified (Song et al., 2015).

### Inversions

**Figure 5:**
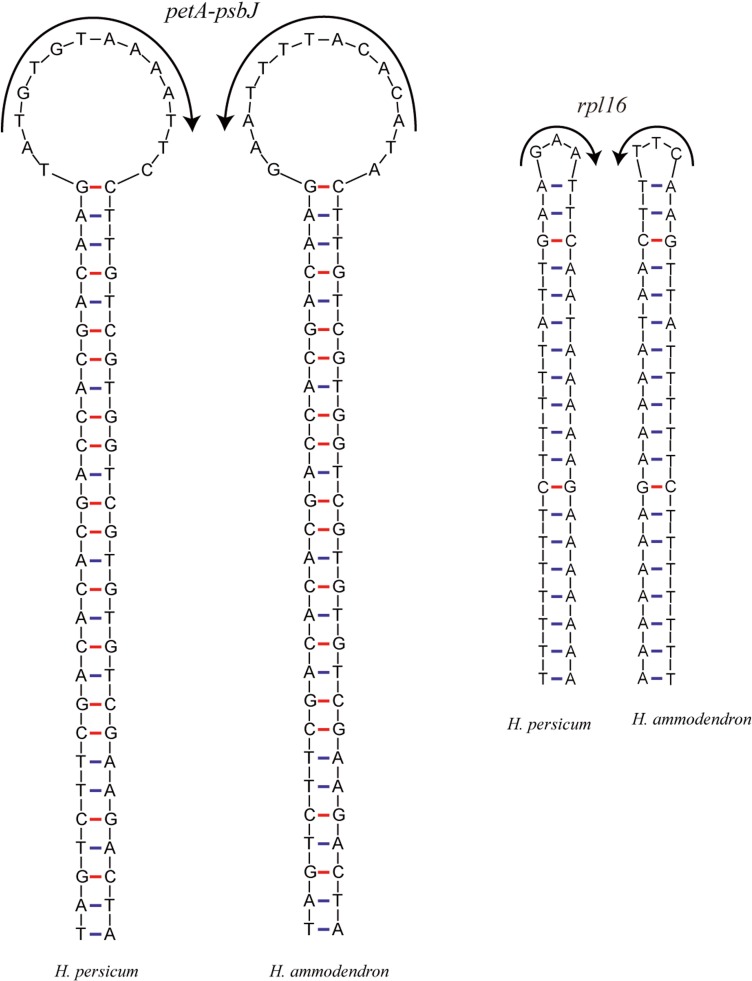
The hairpin loops predicted to be formed by inversions in the *Haloxylon* chloroplast genomes.

Inversions are important events in the evolution of plant cp genomes. Smaller inversions are less frequent in these genomes, and they are generally associated with hairpins (Fig. 5). Most inversions are found in spacers and introns, and in most cases, the presence/absence of inversions is highly homoplastic during cp genome evolution (Kim & Lee, 2005; Catalano, Saidman & Vilardi, 2009), even at the population level (Quandt & Stech, 2004). A sequence alignment of the *Haloxylon* cp genomes revealed that an inversion event of 14 bp and one of 3 bp occur in the *pet*A-*psb*J intergenic region and in the *rpl*16 intron, respectively. The two inverted sequences are predicted to form secondary hairpin structures, with repeat sequences of 27 bp and 19 bp at the two ends, respectively (Fig. 5).

### Pseudogenes

Pseudogenes have been defined as nonfunctional regions of genomic DNA that originally derived from functional genes (Balakirev & Ayala, 2003). These are evolutionary relics of functional components in the genome that provide important information regarding the history of the gene and genome evolution (Balakirev & Ayala, 2003; Zou et al., 2009; Choi & Park, 2015). The *rpl*22 and *rps*18 genes are putative pseudogenes in the Paeoniaceae (Dong et al., 2013b), whereas the *atp*B gene is a pseudogene in *Aster spathulifolius*. Conversely, the *rpl*22, *rps*18, and *atp*B genes are predicted to be normal and functional in the *Haloxylon* species, whereas *rpl*23 is a present as a pseudogene in the *Haloxylon* cp genomes (Fig. 1 and Table S1).

## Conclusions

Two *Haloxylon* cp genomes were sequenced and characterized for the first time, and we found that they share the same overall organization and gene content found in most angiosperm cp genomes, including that of the closely related *Spinacia* and *Beta* species. The location and distribution of repeat sequences and differing nucleotide mutation sites between the two cp genomes were identified. The LSC/IRB/SSC/IRA boundary regions of the Amaranthaceae cp genomes were compared, and lightly intense variations were identified within the genus *Haloxylon*. The complete *Haloxylon* cp genome sequences reported here enhance the genomic information available for the Amaranthaceae family and further contribute to the study of germplasm diversity. These data represent a valuable source of markers for future research on *Haloxylon* population genetics.

##  Supplemental Information

10.7717/peerj.2699/supp-1 Table S1 Genes found in the *Haloxylon* chloroplast genomes

## Additional Information and Declarations

### Field Study Permissions

The following information was supplied relating to field study approvals (i.e., approving body and any reference numbers): Minqin Eremophytes Botanical Garden, Gansu Province, China (under the leadership of Gansu Desert Control Research Institute, Gansu, China). The approval numbers are 2012BAD16B0101 and 80117B1001.

### DNA Deposition

The following information was supplied regarding the deposition of DNA sequences: The chloroplast genome sequences have been deposited to GenBank with the following accession numbers: KF534478 for *Haloxylon ammodendron* and KF534479 for *Haloxylon persicum*.
