# Supplementary material for: Comparative analysis of the complete chloroplast genome sequences in psammophytic Haloxylon species (Amaranthaceae)
Source: PeerJ. 2016 Nov 10;4:e2699. doi: 10.7717/peerj.2699 (PMC5111891; doi:10.7717/peerj.2699)
Supplement: Table S1 [file peerj-04-2699-s001.docx]

**Table S1 Genes found in the *Haloxylon* chloroplast genomes*.***

| **Category for genes** | **Group of gene** | **Name of gene** |
| --- | --- | --- |
| Photosynthesis related genes | Rubisco | *rbc*L |
|  | Photosystem Ⅰ | *psa*A, *psa*B, *psa*C, *psa*I, *psa*J |
|  | Assembly/stability of photosystem Ⅰ | **ycf*3, *ycf*4 |
|  | Photosystem Ⅱ | *psb*A, *psb*B, *psb*C, *psb*D, *psb*E, *psb*F, *psb*H, *psb*I, *psb*J, *psb*K, *psb*L,*psb*N,*psb*T,*psb*Z |
|  | ATP synthase | *atp*A, *atp*B, *atp*E, **atp*F, *atp*H, *atp*I |
|  | cytochrome b/f compelx | *pet*A, **pet*B, **pet*D, *pet*G, *pet*L, *pet*N |
|  | cytochrome c synthesis | *ccs*A |
|  | NADPH dehydrogenase | **ndh*A, **ndh*B, *ndh*C, *ndh*D, *ndh*E, *ndh*F, *ndh*G, *ndh*H, *ndh*I, *ndh*J, *ndh*K |
| Transcription and translation related genes | transcription | *rpo*A, *rpo*B, **rpo*C1, *rpo*C2 |
|  | ribosomal proteins | *rps*2, *rps*3, *rps*4, *rps*7, *rps*8, *rps*11, **rps*12, *rps*14, *rps*15, **rps*16, *rps*18, *rps*19,**rpl*2, *rpl*14, **rpl*16, *rpl*20, *rpl*22, *rpl*32, *rpl*33, *rpl*36 |
|  | translation initiation factor | *inf*A |
| RNA genes | ribosomal RNA | *rrn*5, *rrn*4.5, *rrn*16, *rrn*23 |
|  | transfer RNA | **trn*A-UGC, *trn*C-GCA, *trn*D-GUC, *trn*E-UUC, *trn*F-GAA,*trn*G-GCC, **trn*G-UCC, *trn*H-GUG, *trn*I-CAU, **trn*I-GAU,**trn*K-UUU, *trn*L-CAA, **trn*L-UAA, *trn*L-UAG, *trn*fM-CAU, *trn*M-CAU, *trn*N-GUU, *trn*P-UGG, *trn*Q-UUG, *trn*R-ACG, *trn*R-UCU, *trn*S-GCU, *trn*S-GGA, *trn*S-UGA, *trn*T-GGU, *trn*T-UGU, *trn*V-GAC, **trn*V-UAC, *trn*W-CCA, *trn*Y-GUA |
| Other genes | RNA processing | *mat*K |
|  | carbon metabolism | *cem*A |
|  | fatty acid synthesis | *acc*D |
|  | proteolysis | **clp*P |
| Genes of unknown function | conserved reading frames | *ycf*1, *ycf*2 |
| Pseudogenes |  | *rpl*23, *ycf*15 |

Intron-containing genes are marked by asterisks (*).
